# Adrenocortical Carcinoma: Current Therapeutic State-of-the-Art

**DOI:** 10.1155/2012/234726

**Published:** 2012-10-18

**Authors:** Amir H. Lebastchi, John W. Kunstman, Tobias Carling

**Affiliations:** Department of Surgery, Yale Endocrine Neoplasia Laboratory, Yale School of Medicine, 333 Cedar Street, TMP202, Box 208062, New Haven, CT 06520, USA

## Abstract

Adrenocortical carcinoma (ACC) is a rare, aggressive malignancy that generally conveys a poor prognosis. Currently, surgical resection is considered the lone curative treatment modality. In addition, the low prevalence of ACC has limited effective clinical trial design to develop evidence-based approaches to ACC therapy. The proper role of radio- and chemotherapy treatment for ACC is still being defined. Similarly, the molecular pathogenesis of ACC remains to be fully characterized. Despite these challenges, progress has been made in several areas. After years of refinement, an internationally accepted staging system has been defined. International collaborations have facilitated increasingly robust clinical trials, especially regarding agent choice and patient selection for chemotherapeutics. Genetic array data and molecular profiling have identified new potential targets for rational drug design as well as potential tumor markers and predictors of therapeutic response. However, these advances have not yet been translated into a large outcomes benefit for ACC patients. In this paper, we summarize established therapy for ACC and highlight recent findings in the field that are impacting clinical practice.

## 1. Introduction

Adrenocortical carcinoma (ACC) is a rare, aggressive malignancy that features a correspondingly poor prognosis. The pathogenesis of ACC is poorly understood, especially at the molecular level, as the rarity of the disease makes comprehensive study difficult. As a result, therapeutic options for ACC are currently limited, with medical and radiation therapy remaining complementary to surgery, which is currently the lone curative modality for ACC. Additionally, apart from surgery, treatment for ACC has never been standardized due to the lack of large randomized trials. However, ACC therapy is now evolving. Novel research and the increasing quality of clinical trials may improve available treatment options and outcomes for ACC patients as novel chemotherapeutic agents are introduced and long-standing drug regimens are reassessed.

An overall incidence of 0.5–2 per 1 million cases of ACC have been reported annually worldwide [1, 2]. ACC shows a slight female gender preference and a bimodal age distribution with the first peak in children less than five years of age and the second peak in the fourth to fifth decade of life. Most cases of ACC are sporadic, although some familial cancer syndromes, such as Li-Fraumeni and Beckwith-Wiedemann syndromes, are associated with an increased incidence of ACC [3]. Approximately 60% of patients present with symptoms of excess hormone secretion, most commonly in the form of cortisol hypersecretion (most commonly, hypercortisolism: Cushing's syndrome), with or without virilization due to accompanying androgen excess. Progression is rapid, generally with less than 12 months elapsing from the first clinical changes to advanced Cushing's syndrome [4, 5]. Interestingly, hormonal secretion patterns can vary according to size, differentiation, and stage of the tumor. In cases without clinical hormone overactivity, the most common presentation is related to tumor growth and encroachment on the surrounding viscera, with symptoms such as abdominal discomfort, back pain, and nausea or vomiting. Despite this, overproduction of hormonal precursors is detectable in virtually all cases of ACC, due to defective steroidogenesis within the tumor.

Adrenocortical malignancy can be, regardless of biochemical activity, notoriously difficult to diagnose. In tumors confined to the adrenal gland, the diagnosis may be unclear even after pathological assessment following surgical resection; a widely validated scoring classification (Weiss criteria) is employed in such cases to improve accuracy of diagnosis [6]. On computed tomography (CT), ACC can demonstrate central tumor necrosis, calcifications, and also tends to be larger and more heterogeneous. Reliance on size alone can be misleading, as the widely utilized 4 cm cutoff has a sensitivity of only 81% for ACC. However, ACCs exhibit a significantly higher density on noncontrast CT than adenomas, with a specificity for differentiating adenoma from carcinoma of 100% and 96.9% using 10 and 20 Hounsfield unit cutoffs, respectively [7–9]. Steroid profiling, which is distinct from routine biochemical analysis for adrenal hormone production, is another promising method for differentiating adrenocortical adenomas (ACAs) from ACCs. By using gas chromatography/mass spectrometry to analyze the steroid profiles in 24-hour urine samples of patients with ACCs or ACAs versus control patients, Arlt et al. have identified several metabolites with diagnostic utility. In a retrospective study, their algorithm demonstrated a sensitivity and specificity of 88% for differentiating ACC and ACA when using the nine metabolites identified to have the most diagnostic significance, which exceeds the accuracy of CT alone [10].

Survival for patients with ACC is poor and related to stage at time of diagnosis, which is often advanced. Up to 70% of patients present with extra-adrenal disease [11]. Overall cancer-specific mortality (CSM) rates have been reported between 16% and 38% [1, 12, 13]. Five-year survival for patients with disease confined to the adrenal gland is size-dependent and varies from 61 to 82%. Those with distant metastases at diagnosis have a five-year survival of only 18% [14]. Improved radiographic imaging and surveillance of incidentally discovered adrenal masses have resulted in earlier detection and earlier staging at diagnosis.

## 2. Staging

The TNM staging system is considered the most important tool in prognostic stratification and therapy planning and stratifies cancer patients by survival based on clinical status [2, 12–14]. However, due to the low incidence of ACC and resultant inability to validate ACC staging and survival with any statistical reliability, no TNM classification was available for ACC until recently. Multiple different staging systems were used prior to that time, the most widespread being devised in 1978 by Sullivan, who modified the original McFarlane staging system [2, 15–17]. In 2004, the International Union Against Cancer (UICC) and the World Health Organization (WHO) proposed a new staging system based on Sullivan-McFarlane criteria (Table 1) [18].

To evaluate the UICC system, a large European study examined 416 patients and found a low correlation between five-year disease-specific survival and stage for patients with stage II and III disease. A new staging system was therefore proposed by the European Network for the Study of Adrenal Tumors (ENS@T) consisting of the following major changes.

1. Existence of a thrombus in the inferior vena cava or adjacent renal veins upstages the tumor to T4, with a corresponding grading of stage III.
2. Stage IV tumors are exclusively defined as those with distant metastases.

The ENS@T criteria have been validated in a large North American trial evaluating 573 patients, with a statistically significant difference in cancer-specific mortality now observed between stage II and III patients when the new criteria were applied. Furthermore, 3-year accuracy in predicting CSM rates improved for all patients regardless of stage from 79.5% to 83.0%, when the ENS@T rather than UICC criteria were employed.

## 3. Surgery

### 3.1. Established Therapy

In newly diagnosed cases of ACC, feasibility of surgical resection is the most important contributor to overall survival. While successful treatment of ACC requires a multidisciplinary approach, complete surgical resection is mandatory if possible for patients presenting with stage I to stage III disease. The goal of surgery is R0 resection of the tumor and any involved tissues or viscera in an en bloc fashion. Patients undergoing successful resection have a five-year survival of 40–50%, while median survival of unresectable patients is less than one year [17, 34, 35]. When stratified by stage at time of resection, 5-year disease-specific survival was found to be 82% for stage I disease, 58% for stage II, 55% for stage III, and 18% for stage IV [14].

There is consensus that resection should be performed by an experienced multidisciplinary team [36]. This is especially crucial in the management of patients with biochemically active tumors. Intraoperatively, maintaining tumor capsule integrity and preventing tumor spillage are key considerations [4]. Notably, presence of tumor thrombi and vascular invasion are not contraindications to resection. In cases with extensive vascular involvement, usage of cardiopulmonary bypass can facilitate successful resection [37]. A transabdominal, open surgical approach allows maximal exposure. This facilitates en bloc excision of tumor and other involved organs, maintenance of the tumor capsule, and effective vascular control when necessary.

There is an ongoing debate on the role of lymphadenectomy. A retrospective analysis of the data from the German ACC registry by Reibetanz et al. indicated that locoregional lymph node dissection improved tumor staging and lead to a favorable oncologic outcome in patients with localized ACC [38]. For recurrent disease, reoperation with the goal of radical resection or tumor debulking is beneficial in those patients who are surgical candidates [35, 39]. During reoperation, complete resection is again crucial, resulting in a mean survival time of 74 months versus 16 months in those with incomplete resections [35]. In some patients that display unresectable disease at the time of diagnosis, debulking may be beneficial in tumors [36]. Debulking may provide relief from symptoms of hormonal excess and facilitate additional treatment options [20, 40]. Conversely, patients with widely metastatic disease or rapidly enlarging tumors at diagnosis are better managed with medical palliation only.

### 3.2. Emerging Trends

Since its introduction in 1992, laparoscopic adrenalectomy (LA) has become the treatment of choice for benign adrenal tumors due to improvements in postoperative analgesia use, cosmesis, and length of hospital stay [41, 42]. Resection of ACC via a laparoscopic approach, while technically feasible, remains highly controversial. Initial reports evaluating laparoscopic versus open adrenalectomy in ACC patients noted both higher rates of recurrence and shorter disease-free survival in those with laparoscopic resections [19, 33]. Notably, those undergoing laparoscopic resection demonstrated a substantially increased rate of local recurrence and peritoneal carcinomatosis compared to recurrences in those undergoing open resection (83% versus 43% in one study), suggesting loss of capsule integrity and port site seeding as potential causes of the poorer outcomes seen with laparoscopy [19]. Conversely, several more recent studies have found comparable outcomes between laparoscopic and open approaches in ACC resection (Tables 2 and 3). One study evaluating 152 patients undergoing either laparoscopic (*n* = 35) or open (*n* = 117) adrenalectomy for ACC found identical oncologic outcomes. However, it was limited to patients with tumor size ≤ 10 cm and 12 patients undergoing laparoscopic resection required conversion to an open approach [32]. An Italian study demonstrated similar findings in a cohort limited to patients with a stage I and II disease only [43]. In general, superior surgical outcomes following adrenalectomy are observed in centers with high-volume surgeons and considerable expertise; this is especially true in adrenalectomy for ACC [44, 45].

## 4. Radiotherapy

### 4.1. Established Therapy

Historically, radiation therapy (RT) has not been considered effective in treatment of primary ACC [46–49]. ACC is not an overly radiosensitive tumor, and the anatomical proximity to radiosensitive viscera such as the small bowel, kidney, and spinal cord has limited the clinical utility of radiotherapy. There is a well-defined role for RT in the treatment of metastatic ACC, especially in bony disease [50, 51]. Furthermore, radiotherapy can improve symptoms in patients with bulky abdominal tumors that are unresectable [52]. In a study of 91 patients in the German ACC registry, a response rate of 57% was noted in patients receiving palliative radiotherapy [53], and an investigation of the Dutch ACC registry showed that ACC can be radiosensitive and patients with advanced disease can benefit from it [54].

### 4.2. Emerging Techniques

The role of radiotherapy in an adjuvant therapy for ACC remains controversial. Improvements in technology and radiotherapy protocols (specifically, stereotactic body radiation therapy) have resulted in superior morbidity profiles compared to historical controls due to improved targeting and lower nontumor dosing. However, there is still no prospective evidence supporting radiotherapy in an adjuvant setting. The best current evidence advocates for the usage of radiotherapy for local control in unresectable disease and after resection in certain cases [55]. In general, adjuvant radiotherapy has been advocated as a means to reduce the high incidence of local recurrence observed in ACC. A recent North American study evaluating surgery alone versus surgery and radiotherapy found that those treated with surgery alone had an odds ratio for local recurrence of 4.7 versus those who received adjuvant radiation [56]. These data concur with a European study that also found increased local recurrence in the surgery alone group [57]. However, that study failed to demonstrate either a disease-free or overall survival benefit.

 The lack of strong evidence supporting radiotherapy has resulted in a variety of treatment recommendations. The German ACC group currently recommends radiotherapy in the following cases [53]:

1. all patients with incomplete (R1 or R2) or uncertain (Rx) resections,
2. all patients with stage III disease regardless of resection adequacy,
3. strong consideration in cases of >8 cm tumor size, Ki-67 index > 10%, and invasion of adjacent vasculature, even in cases of complete resection.

Prospective data are needed to fully define the role of radiotherapy in the adjuvant setting for ACC. Despite this, the available evidence supports treatment in patients with incomplete resection, stage III disease, or in the palliative setting.

## 5. Medical Therapy

Medical therapy for ACC takes two forms. Cytotoxic agents, of which mitotane (1,1-dichloro-2(*o*-chlorophenyl)-2-(*p*-chlorophenyl)ethane) is the prototype, have been the mainstay of ACC chemotherapeutics for decades and have been studied in adjuvant, recurrent, and palliative settings. Disagreement persists over the most efficacious treatment regimen in each role. Biologic pharmaceuticals have recently been introduced into practice for ACC treatment based on rational selection of molecular targets. Numerous biologic agents are currently being evaluated in clinical trials.

## 6. Adjuvant Systemic Therapy

Mitotane is a derivate of the insecticide DDT and is directly toxic to the adrenocortical parenchyma. It has been the mainstay of systemic ACC treatment since the 1960s. Clinical response to mitotane is not universal, possibly due to the need for metabolic transformation of mitotane for therapeutic action [58]. The therapeutic index of mitotane is narrow; up to 80% of patients develop side effects, some of which can lead to cessation of therapy [59]. Nausea, emesis, and other gastrointestinal symptoms are most common, but neurologic toxicity can occur as well, especially at high dose ranges. A serum concentration of 14–20 mcg/mL is usually considered therapeutic [13, 60–62]. Despite this, the optimum dose regimen is unknown. A therapeutic range can be achieved with a low-dose scheme designed to minimize toxicity [21]. However, most recurrences in the adjuvant setting occur less than six months postoperatively and the time to achieve effective serum levels is increased when utilizing a low-dose regimen [63, 64].

 Currently, no prospective clinical trials exist evaluating adjuvant mitotane use. Several earlier observational studies did not report improved overall or disease-free survival with adjuvant mitotane use [65, 66], while more recent studies have noted a modest benefit [23, 67]. The best available evidence comes from a large European retrospective study evaluating 177 patients with ACC, with 47 patients receiving surgery and adjuvant mitotane and 130 receiving surgery alone. Recurrence-free survival was significantly improved in the treatment group. However, the surgery alone group had a higher incidence of advanced disease, which the authors controlled for by employing a multivariate statistical model which continued to demonstrate a benefit to mitotane use [22]. As such, the decision to administer adjuvant mitotane remains controversial. A panel of international experts in 2008 unanimously recommended adjuvant mitotane in patients with potential residual disease (R1 or Rx resection) or greater than 10% Ki67 positivity on pathologic examination [68]. Similarly, the same panel did not consider mitotane to be mandatory to patients with stage I or II disease who underwent histologically proven R0 resection with Ki67 indices less than 10%. The panel was undecided on whether to offer adjuvant therapy to stage III ACC patients following an R0 resection. Currently, a prospective randomized trial evaluating adjuvant mitotane use is recruiting patients in several European centers with the goal of improving future treatment algorithms.

## 7. Systemic Therapy in Locally Advanced and Metastasized Disease

Standard of treatment in patients with unresectable or metastasized ACC previously consisted of mitotane alone or in combination with other cytotoxic drugs [36, 69]. Generally, prognosis is poor in this patient population, although reports of long-term survival exist [35, 70, 71]; it is unclear whether this is due to favorable tumor biology or therapeutic intervention. Studies investigating mitotane alone in these patients have demonstrated a response rate of 19–33%, but with minimal survival benefit in responders (9 months in the largest trial) [60, 62, 72]. Mitotane in combination with standard cytotoxic agents has been investigated, with the two most popular regimens of mitotane-streptozocin (M-Sz) and mitotane etoposide/doxorubicin/cisplatin (M-EDP) having previously been investigated in phase II trials. In separate studies, an objective responsive rate of 36% was observed in 22 patients with advanced ACC receiving M-Sz [73] and in 53% of 28 patients receiving M-EDP [74]. Recently, the FIRM-ACT (First International Randomized Trial in Locally Advanced and Metastatic ACC Treatment) trial was released, which is a landmark randomized controlled trial comparing M-Sz and M-EDP in 304 patients with advanced ACC [75]. The trial found M-EDP statistically superior to M-Sz in terms of objective tumor response (23.2 versus 9.2%, resp.), progression-free survival (5.0 versus 2.1 months), and proportion of patients without progression at one year (23.2% versus 9.2%). Tumor burden was monitored by serial CT scans every eight weeks. Overall survival at the time of the study's conclusion favored M-EDP (14.8 months versus 12.0 months) without reaching statistical significance. The study also included an elegantly designed nested trial evaluating each regimen as a second-line regimen, whereas patients with treatment failure in their original group were given the alternative regimen (but still underwent primary endpoint analysis in an intent-to-treat manner). As second-line therapies, the efficacy of both regimens was similar to their efficacy as first-line therapy, with M-EDP showing superior antitumor efficacy and progression-free survival. Notably, the authors found no statistically significant difference in quality of life or rate of adverse events between the two treatment groups. As such, selection of systemic treatment for recurrent or metastatic ACC should favor M-EDP as the first-line therapy of choice in the future.

## 8. Targeted Therapy

Ongoing research into the oncogenesis of ACC has increased knowledge of the molecular mechanisms of ACC tumor growth and also identified potential targets for drug development. Gene transcriptome analysis has elucidated significant differences between the gene expression profile of ACC and benign adrenocortical adenomas with a large number of genes demonstrating differential or tumor-specific expression [76–80]. Szabó et al. analyzed microarray data from several studies and found a significant upregulation of genes involved in the cell cycle, growth factors and receptors and, simultaneously, a downregulation of genes that play a role in steroidogenesis, metabolism, and cell transport in ACC compared to benign disease [81]. Alterations in expression of the insulin-growth factor genes (IGF-1 and -2) are one of the most common mutations in ACC and one of the earliest recognized [82, 83]. Furthermore, additional growth factors have been implicated in ACC such as epidermal growth factor (EGF), transforming growth factor-*α* (TGF-*α*), and vascular endothelial growth factor (VEGF). Many of the receptors for these ligands belong to the tyrosine kinases receptor superfamily, inhibitors of which are already in clinical use for other malignancies [84]. In general, initial results utilizing tyrosine kinase inhibitors and other targeted therapeutics for ACC have been unable to improve upon the current standard of care as yet [4, 85–88]. The following section summarizes the current experience with selected agents that are in clinical investigation or have been promising in preclinical studies. Active clinical trials utilizing targeted therapy for ACC are listed in Table 4.

### 8.1. IGF Antagonists

IGF-1 and -2 are implicated in ACC development through both the phosphoinositide-3-kinase (PI3 K)-Akt and the Ras-Raf-MAP kinase pathways. Genome-wide gene expression studies have identified that overexpression of IGF-2, the ligand to the IGF-1 receptor which activates both of the above pathways, is a very consistent molecular event in the development of ACC [76, 77, 82, 89, 90]. Haluska et al. investigated an anti-IGF-1R antibody (figitumumab) in a phase I study and found it to be well tolerated and achieved stability of disease in 8 out of 14 patients (57%) [91]. The GALACCTIC trial is a phase 3 randomized, double-blinded trial investigating OSI-906, a combined inhibitor of IGF-1R and insulin receptor(IR) in patients with locally advanced or metastatic ACC who failed at least one prior drug regimen. OSI-906 has shown an antineoplastic effect in vitro [92]. The study has reached its accrual goals and is currently ongoing.

### 8.2. mTOR Antagonists

The mammalian target of rapamycin (mTOR) is a serine/threonine protein kinase that is involved in cell growth and proliferation that is activated in part by IGF-1R signaling via the aforementioned PI3 K-Akt pathway. The prototype mTOR inhibitor, sirolimus, has long been utilized as an antirejection drug following solid organ transplant, and mTOR inhibitors have recently been investigated in clinical trials for treating malignancies, such as renal cell carcinoma [93]. Given its association with the IGF-1R pathway known to be dysregulated in ACC, inhibition of mTOR seems an appropriate target for investigation in ACC treatment. Doghman et al. demonstrated that inhibition of mTOR signaling reduced adrenocortical tumor growth in vitro and in an in vivo mouse model in 2010 [94]. Temsirolimus, a second-generation mTOR inhibitor in combination with cixutumumab, an anti-IGF-1R antibody, has been welltolerated in phase I trials in patients with advanced tumors, including ACC [95]. Additional clinical trials are ongoing (see Table 4).

### 8.3. Future Targets

The canonical Wnt/*β*-catenin signaling pathway, involved in both human development and homeostasis, is dysregulated in a large number of human disease processes [96]. Microarray analysis has demonstrated upregulation of this pathway in ACC, and *CTNNB1*, the gene encoding *β*-catenin, is frequently mutated in adrenocortical neoplasia [97, 98]. Furthermore, *CTNNB1, *the gene encoding *β*-catenin, has been found to be frequently mutated in both ACC and adrenocortical adenomas [99]. This common mutation in both benign and malignant adrenocortical neoplasia may indicate an early step in a common pathway of tumorigenesis. Berthon et al. demonstrated a clear link between constitutive *β*-catenin activation and adrenal cortex dysplasia in a transgenic murine model, resulting in malignant changes such as neovascularization and local tumor invasion [100]. However, drugs effectively targeting the Wnt pathway have been slow to develop and further research identifying specific genetic targets is needed.

Steroidogenic factor (SF)-1 is an orphan nuclear receptor that has a key role in normal endocrine and gonadal development, as well as regulating steroid production in the adrenal cortex via interactions with several cytochrome P450 steroid hydroxylases in the adrenal cortex [101]. Overexpression of SF-1 has been demonstrated in ACC, especially in pediatric cases [102, 103]. Interestingly, SF-1 has been demonstrated to have a dose-dependent effect on the induction of adrenocortical cell proliferation through changes in apoptosis and cell cycle control [104]. In a mouse model, SF-1 induced adrenal hyperplasia and tumor growth. Antagonists of SF-1 inverse agonists have been identified and demonstrated inhibition of adrenocortical cell proliferation and steroidogenesis [105].

### 8.4. Individualized Treatment

Giordano et al. first reported transcriptional profiling of ACC in 2003. Genetic profiling of individual tumors has many potential benefits, but especially relevant to treatment of ACC is the opportunity to predict response rates to certain pharmaceutical agents and determination of prognosis. Aside from improving patient care, a secondary benefit of such information would be segregation of patients into clinical trial subgroups with others that have their specific form of disease [97]. Several molecular markers of drug sensitivity in ACC have been identified and are under investigation, as standard ACC chemotherapy is associated with significant toxicity and variable response rates characteristics [106]. For instance, ERCC1, a DNA repair protein, has previously been shown to predict resistance to platinum-based chemotherapy regimens. Less than 50% of ACCs treated with a platinum-based regimen shows a clinical response, and ACC patients demonstrating a high rate of ERCC1 expression following platinum-based chemotherapy have a significantly shortened median survival time (8 versus 24 months in low-ERCC1 patients) [107]. Additionally, a phase II trial is underway for ACC utilizing a regimen including XR9576, an inhibitor of MDR1, expression of which has been implicated in other multidrug-resistant tumors [108, 109]. Ideally, as the cost of genetic sequencing continues to fall and knowledge of the implications of specific genetic changes on treatment response and outcome improves, treatment regimens can be optimized on an individualized basis.

## 9. Conclusion

ACC remains a rare malignancy that has seen little improvement in overall mortality over the past two decades. Until recently, standard of care was based only on individual opinion and occasionally expert consensus. Over the past decade, international collaboration has begun to improve the management of these patients and the molecular understanding of the disease. The development of standardized staging criteria and the gradual accrual of clinical trials in treating ACC, especially with the release of the first randomized phase III trial in ACC, exemplifies the ongoing progress in clinical care of these patients. Similarly, as the genetic understanding of adrenal tumorigenesis continues to progress, more targets for future drug development are identified and evaluated. As these discoveries are translated into clinical practice, ACC therapy will move beyond historical modalities to the benefit of patients everywhere.

## Figures and Tables

**Table 1:** Comparison UICC and ENSAT staging systems for ACC.

Stage	UICC/WHO 2004	ENSAT 2008
I	T1, N0, M0	T1, N0, M0

II	T2, N0, M0	T2, N0, M0

III	T1-2, N1, M0	T1-2, N1, M0
	T3, N0, M0	T3-4, N0-1, M0

	T1–4, N0-1, M1	T1–4, N0-1, M1
IV	T3, N1, M0	
	T4, N0-1, M0	

T1: tumor ≤ 5 cm; T2: tumor > 5 cm; T3: tumor infiltration in surrounding tissue; T4: tumor infiltration in adjacent organs [ENSAT additionally the presence of a tumor thrombus in the Vena Cava or Vena Renalis]; N0: absence of positive lymph nodes; N1: presence of positive lymph nodes; M0: absence of distant metastases; M1: presence of distant metastasis.

**Table 2:** Retrospective series of open adrenalectomies.

Author	Total number of malignant cases	Mean tumor size (cm)	Duration of followup (months)	Recurrence rate	Comments
Gonzalez et al., 2005 [19]	133 ACC	NR	28	51%	Median survival duration: 34 months
Crucitti et al., 1996 [20]	91 ACC	NR	NR	15%	Median survival duration: 28 months
Terzolo et al. [21, 22]	55 ACC	10	67	90	Median survival duration: 52 months
	75 ACC	10	43	73	Median survival duration: 67 months
Icard et al., 2001 [17]	253 ACC	12	NR	NR	5 year survival: 38%
Kendrick et al., 2001 [23]	58 ACC	12.5	53	51	5 year survival: 37%

**Table 3:** Summary of series of laparoscopic adrenalectomies for adrenal malignancies.

Author	Total number of LA	Total number of malignant cases	Mean tumor size (cm)	Duration of followup (months)	Recurrence rate	Comments
Henry et al., 2002 [24]	233	6 ACC	7.4	47	17%	1 dead of disease
Porpiglia et al., 2004 [25]	205	6 ACC	6.9	30	0%	1 dead of cerebrovascular accident
Corcione et al., 2005 [26]	100	2 ACC	8.5	13.6	50%	Both patients alive, one has still disease
*Gonzalez et al., 2005* [19]	*6 *	*6 ACC *	*5.3 *	*28 *	*100% *	*2 are still alive with disease, remaining 4 died of disease *
Palazzo et al., 2006 [27]	391	3 MP	6.8	34	33%	1 dead of disease
Lombardi et al., 2006 [28]	79	4 ACC 3 MP	5.9	23	29%	4 alive disease free, 2 alive with disease and one dead of liver failure
Liao et al., 2006 [29]	210	4 ACC	6.2	39	25%	1 alive disease free, 1 alive with disease and 2 died of disease
Nocca et al., 2007 [30]	131	4 ACC	8.5	34	25%	3 alive disease free, 1 died of metastatic disease
Ramacciato et al., 2008 [31]	18	2 ACC	8.3	44	0%	Alive and disease free
Brix et al., 2010 [32]	35	35 ACC	6.2	39	77%	37% of patients died from ACC
Miller et al., 2010 [33]	17	17	7.0	36	20%	Not investigated, but authors concluded that the mean time to local recurrence was shorter in LA compared to the open group

ACC: adrenocortical cancer; MP: malignant pheochromocytoma; LA: laparoscopic adrenalectomy.

**Table 4:** Ongoing Clinical Trials that test the Target Therapies.

Study	Target	ID	Purpose	Status
Mitotane with or without *IMC-A12* in treating patients with recurrent, metastatic, or primary adrenocortical cancer that cannot be removed by surgery	*IGF1R *	NCT00778817	This randomized phase II trial compares the combination of mitotane and IMC-A12 with mitotane alone in the treatment of recurrent, metastatic, or primary adrenocortical cancer that cannot be removed by surgery	Recruiting

A study of *OSI-906* in patients with locally advanced or metastatic adrenocortical carcinoma (GALACCTIC)	*IGF1R *	NCT00924989	A multicenter, randomized, double-blind, placebo-controlled, phase III study of single-agent OSI-906 in patients with locally advanced/metastatic adrenocortical carcinoma who received at least 1 but no more than 2 prior drug regimens	Ongoing not recruiting

Phase II trial of *ZD1839* (*Iressa*) in patients with nonresectable adrenocortical carcinoma	*VEGFR *	NCT00215202	This phase II trial investigates the effect of Iressa in patients with nonresectable adrenocortical cancer who have previously been treated with one other form of systemic therapy (either Mitotane or chemotherapy).	Completed

Phase II Study of *Axitinib* (AG-013736) With Evaluation of the VEGF-Pathway in Metastatic, Recurrent or Primary Unresectable Adrenocortical Cancer	*Multikinase *	NCT01255137	To evaluate the effectiveness of axitinib in individuals who have adrenocortical cancer that is inoperable and has not responded to standard treatments	Recruiting
	(i) *VEGFR *			
	(ii) *PDGFR *			
	(iii) *KIT *			

*Sunitinib* in Refractory Adrenocortical Carcinoma (SIRAC)	*Multikinase *	NCT00453895	The primary objective of this trial is to estimate the response (defined as progression-free survival of ≥12 weeks) rate associated with Sunitinib treatment in patients advanced ACC progressing after cytotoxic chemotherapy	Unknown
	(i) *VEGFR *			
	(ii) *PDGFR *			
	(iii) *KIT *			

*Sorafenib* Plus Paclitaxel in adreno-cortical-cancer patients (PAXO)	*Multikinase *	NCT00786110	The aim of this phase II trial is to evaluate the clinical benefit and toxicity of the combination of Sorafenib plus metronomic chemotherapy in patients with locally advanced or metastatic ACC who progressed after first or second line chemotherapy.	Unknown
	(i) *RAF *			
	(ii) *VEGFR *			
	(iii) *PDGFR *			
	(iv) *KIT *			

Clinical trial of *Dovitinib* in first-line metastatic or locally advanced non-resectable adrenocortical carcinom	*FGFRs *	NCT01514526	Non-randomized, phase II clinical trial, that investigates the use of Dovitinib in adult patients with metastatic or locally advanced non-resectable adrenocortical carcinoma, confirmed histologically	Recruiting

*Cixutumumab* in treating patients with relapsed or refractory solid tumors	*IGF1R *	NCT00831844	Phase II trial that studies the side effects and how well cixutumumab works in treating patients with relapsed or refractory solid tumors, including ACC	Recruiting
